# The Sleep-Heart-Brain Connection: Sex-Specific Modifiable Risk Factors and Cognitive Impact in Mid-to-Late Life

**DOI:** 10.1093/geroni/igaf122.487

**Published:** 2025-12-31

**Authors:** Ashley Curtis, Amy Costa, Natasa Billeci

**Affiliations:** University of South Florida, Tampa, Florida, United States; University of South Florida, Tampa, Florida, United States; University of South Florida, Tampa, Florida, United States

## Abstract

Poor sleep and cardiovascular health are considered modifiable risk factors of cognitive decline. How these functions interact in associations with cognition, and whether there are sex differences in patterns of association remains underexplored. The present study evaluated sex-specific interactive patterns of sleep health and cardiovascular risk factors on cognition in mid-to-late life. Middle-aged and older adults (N = 270; 124 women; Mage=64.5±7.8) completed measures of sleep health [(Pittsburgh Sleep Quality Index (PSQI)], medical history and demographics (including biological sex, BMI), substance use (Alcohol Use Identification Test, smoking history), and self-reported cognition (Cognitive Failures Questionnaire). A subset of participants (n = 67) completed objective cognitive tasks (Posner Cueing Task, Sternberg Working Memory Task, Stroop Task). Moderation analyses examined sex differences in interactive associations between sleep health, cardiovascular disease (CVD) risk score (combined score including presence of hypertension, cancer, diabetes, smoking, hazardous alcohol use, overweight/obesity) and cognitive function, controlling for age. Sex moderated interactive associations between PSQI-Total, CVD risk score and endogenous attentional orienting. Specifically, in men, greater CVD risk score was associated with worse attentional orienting only in those with worst global sleep quality (PSQI-Total ∼11; p=.02), and this pattern was not observed in women. Worse sleep health may exacerbate the impact of cardiovascular disease risk profile on objective spatial attention in mid-to-late life men. Sex-specific monitoring and tailoring of treatments for cardiovascular and sleep health is encouraged. Prospective studies that examine underlying mechanisms (e.g., circulating testosterone) are needed to understanding sex and sleep-specific interactive risk factors of cognitive decline.

